# Increased prevalence of autoimmune thyroid disease after COVID-19: A single-center, prospective study

**DOI:** 10.3389/fendo.2023.1126683

**Published:** 2023-03-08

**Authors:** Alessandro Rossini, Sara Cassibba, Francesca Perticone, Simone Vasilij Benatti, Serena Venturelli, Greta Carioli, Arianna Ghirardi, Marco Rizzi, Tiziano Barbui, Roberto Trevisan, Silvia Ippolito

**Affiliations:** ^1^ Endocrinology and Diabetes Unit, Papa Giovanni XXIII Hospital, Bergamo, Italy; ^2^ Endocrinology Unit, San Raffaele Scientific Institute, Milan, Italy; ^3^ Infectious Diseases Unit, Papa Giovanni XXII Hospital, Bergamo, Italy; ^4^ FROM Research Foundation, Papa Giovanni XXIII Hospital, Bergamo, Italy

**Keywords:** SARS-CoV-2, COVID-19, thyroid peroxidase (TPO) antibodies, thyroiditis, autoimmune diseases, thyroid dysfunctions, thyroid autoimmunity, autoimmune thyroid disease

## Abstract

**Introduction:**

Thyroid dysfunctions associated with SARS-CoV-2 acute infection have been extensively described since the beginning of COVID-19 pandemics. Conversely, few data are available on the occurrence of thyroid autoimmunity after COVID-19 resolution. We assessed the prevalence of autoimmune thyroid disease (ATD) and thyroid dysfunctions in COVID-19 survivors three months after hospital admission.

**Design and methods:**

Single-center, prospective, observational, cohort study performed at ASST Papa Giovanni XXIII Hospital, Bergamo, Italy. 599 COVID-19 survivors were prospectively evaluated for thyroid function and autoimmunity thyroperoxidase antibodies (TPOAb), thyroglobulin antibodies (TgAb). When a positive antibody concentration was detected, thyroid ultrasound was performed. Multiple logistic regression model was used to estimate the association between autoimmunity and demographic characteristics, respiratory support, and comorbidities. Autoimmunity results were compared to a cohort of 498 controls referred to our Institution for non-thyroid diseases before the pandemic onset. A sensitivity analysis comparing 330 COVID-19 patients with 330 age and sex-matched controls was performed.

**Results:**

Univariate and multivariate analysis found that female sex was positively associated (OR 2.01, SE 0.48, p = 0.003), and type 2 diabetes (T2DM) was negatively associated (OR 0.36, SE 0.16, p = 0.025) with thyroid autoimmunity; hospitalization, ICU admission, respiratory support, or COVID-19 treatment were not associated with thyroid autoimmunity (p > 0.05). TPOAb prevalence was greater in COVID-19 survivors than in controls: 15.7% vs 7.7%, p = 0.002. Ultrasonographic features of thyroiditis were present in 94.9% of the evaluated patients with positive antibodies. TSH was within the normal range in 95% of patients.

**Conclusions:**

Autoimmune thyroid disease prevalence in COVID-19 survivors was doubled as compared to age and sex-matched controls, suggesting a role of SARS-CoV-2 in eliciting thyroid autoimmunity.

## 1 Introduction

Viral infections may trigger autoimmune diseases (1). Reports of autoimmune conditions occurring after SARS-CoV-2 infection have been described (2), including anecdotal cases of Graves’ disease (3–5) and Hashimoto’s thyroiditis (6, 7). However, only few studies systematically evaluated the impact of COVID-19 in the development of autoimmune thyroid disease (ATD). Anaya et al. (8) found an increased prevalence of thyroperoxidase antibodies (TPOAb) in 120 patients hospitalized for COVID-19 as compared to healthy, pre-pandemic controls, suggesting an activation of thyroid autoimmunity by SARS-CoV-2. Consistently, Lui et al. (9) reported an increase in TPOAb concentration in COVID-19 survivors three months after hospital admission. However, most patients in this cohort were treated with interferon beta (IFN-beta) that has been associated per se with the induction of thyroid autoimmunity; the reassessment of a larger cohort of patients not exposed to IFN-beta was thus advocated by the Authors to provide a conclusive answer.

Alterations of thyroid function tests (TFTs) during the acute phase of COVID-19 have been more extensively characterized since the beginning of the pandemics (10, 11). Low TSH levels, attributed either to a destructive thyroiditis associated with thyrotoxicosis or to a non-thyroidal illness (NTI), were reported in several studies (12–18). According to most studies (12–14, 16, 19), TFTs usually normalize after COVID-19 recovery, but this finding has not been established in a large population.

Aim of our study was to assess the prevalence of ATD and thyroid dysfunction in a large cohort of COVID-19 survivors at a medium-term (three months) follow-up after hospitalization.

## 2 Materials and methods

### 2.1 Study cohort

COVID-19 survivors participating to our outpatient service program were eligible for the study. The enrollment protocol has been described in a previous paper (20). Briefly, a list of all patients with COVID-19 discharged from the emergency department or admitted to the hospital wards of our Institution (ASST Papa Giovanni XXIII, Bergamo, Italy) was obtained from the hospital electronic health records database. Asymptomatic positive patients admitted for planned procedures were excluded. Other exclusion criteria were: age less than 18 years, pregnancy, history of thyroid disease or previous thyroid surgery, concomitant medications known to interfere with thyroid function (lithium, amiodarone, interferon-α and antiretroviral drugs), severe kidney insufficiency (eGFR < 30 ml/min), and severe liver failure. Patients’ enrollment took place between 2 May and 31 July 2020, before availability of SARS-CoV-2 vaccines, to avoid potential biases due to occurrence of post-vaccination thyroid disorders (21, 22).

To compare thyroid autoimmunity data, a control group was retrieved from the hospital electronic health records database. Controls were included if i) had one assessment of TPOAb and/or thyroglobulin antibodies (TgAb) from January 2016 to January 2020, ii) their medical history was negative for thyroid disease, and iii) they referred to our Institution for reasons other than a suspected thyroid disease.

### 2.2 Assays

Thyroid stimulating hormone (TSH), TPOAb, and TgAb were measured in all patients; free thyroxine (fT4) and free tri-iodothyronine (fT3) were measured in patients with abnormal TSH levels. A chemiluminescent immunoassay (Atellica Solution, Siemens) was employed. Normal range for TSH, fT4 and fT3 were 0.5-5.0 mIU/L, 0.7-1.8 ng/dL, and 2.3-4.5 pg/mL, respectively. For TPOAb, measuring interval was 28-1300 U/mL and range of normality was below 60 U/mL. For TgAb, measuring interval was 15-500 U/mL and range of normality was below 60 U/mL.

### 2.3 Ultrasound assessment

When positive antibodies were detected, ultrasonography of the thyroid was prescribed. Thyroid volume was calculated with the ellipsoid formula (23): width (mm) x length x thickness x 0.52 = volume (mL) for each lobe. Ultrasonographic diagnosis of thyroiditis was made if one or more of the following features were present: hypoechogenity of gland parenchyma, non-homogeneous parenchymal texture, and increased vascularity. All thyroid ultrasound examinations were performed by two operators (AR and SC) with the same instrument (My Lab Seven, Esaote, Italy), using a 3- to 13-MHz linear transducer.

### 2.4 Statistical analysis

Descriptive statistics was used to summarize clinical characteristics of COVID-19 patients during the acute phase of the disease and at the subsequent clinical evaluation. Continuous variables were expressed as medians and interquartile ranges (IQRs) and categorical variables were presented as frequencies and percentages. The study population was then stratified based on the presence of thyroid autoimmunity (yes/no), and differences between groups were tested using the Mann-Whitney test for continuous variables and the chi-square test (or Fisher’s exact test when appropriate) for categorical variables. To evaluate the association of thyroid autoimmunity and COVID-19, we conducted a sensitivity analysis comparing 330 COVID-19 patients with 330 age and sex-matched subjects retrieved from control group.

A multiple logistic regression model was used to estimate odds ratios (ORs) of autoimmunity and their corresponding 95% confidence intervals (CIs) for the following variables: age (at entrance), sex, respiratory support (no support/low need/high need), and diabetes mellitus (yes/no). In the multivariable analysis were included demographic characteristics, respiratory support (as proxy of disease severity) and covariates that resulted significantly different between groups in the univariate analysis. For all tested hypotheses, two-sided p-values of 0.05 or less were considered significant. Statistical analysis was performed using STATA Software, release 16.1 (StataCorp LP, College Station TX, USA) and was carried out at the biostatistical laboratory of the Foundation for Research (FROM) at Papa Giovanni XXIII Hospital in Bergamo.

## 3 Results

**Figure 1:**
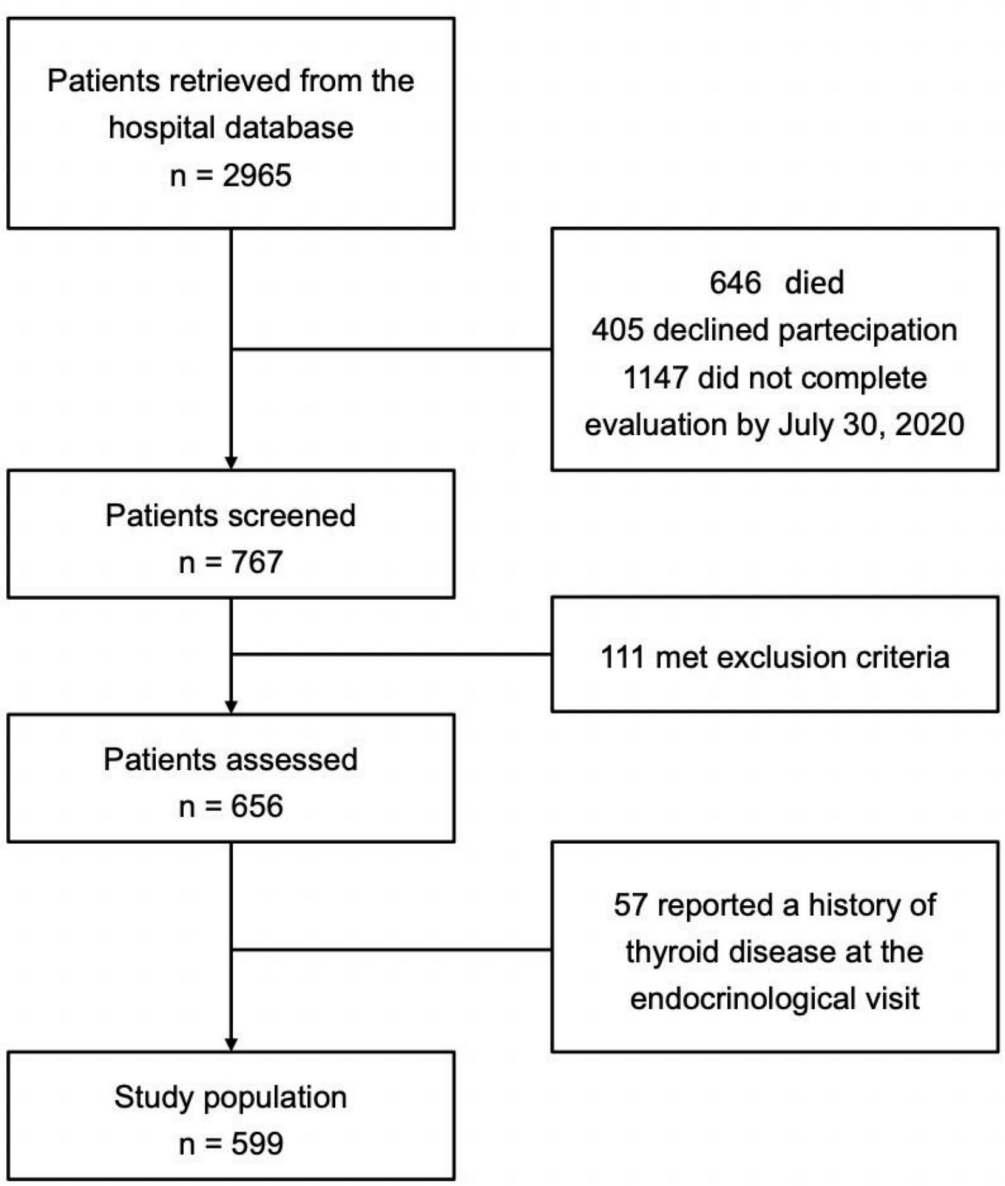
Flow chart of screened, included, and excluded patients.

The search in hospital electronic health records database identified 2965 patients eligible for the study (946 discharged from emergency department and 2019 admitted to Hospital), of which 646 died before the enrollment and 405 declined to participate. Of the remaining 1914, 767 were screened by 31 July 2020. In total, 168 patients met the exclusion criteria for this study. The final population therefore consisted of 599 patients (180 females). **Figure 1** shows the flow-chart describing screened, included, and excluded subjects. Median time at evaluation was 102.5 days after hospital admission.

TPOAb were above the normal range in 85 patients (14.2%), TgAb in 43 (7.2%) and both antibodies in 23 (3.8%) patients. At least one antibody was positive in 105 patients (48 females), with an overall prevalence of thyroid autoimmunity of 17.5%.

Median TPOAb was 102 U/mL (IQR 68.5 – 611) in patients with positive TPOAb and 36 U/mL (IQR 27-44) in patients with negative TPOAb; median TgAb was 174 U/mL (IQR 89.5 – 285.7) in patients with positive TgAb and 18 U/mL (IQR 14-23) in patients with negative TgAb.

Median TSH was 1.55 mIU/L (IQR 1.09 - 2.15); thirty patients (5.0%) showed abnormal TSH values, of which 19 (3.2%) had values below 0.5 mIU/L and 11 (1.8%) above 5.0 mIU/L. All patients with TSH levels < 0.5 mIU/L had normal fT3 and fT4 levels. Nine out of the eleven patients with TSH levels > 5.0 mIU/L had normal fT4 levels, exhibiting a condition of subclinical hypothyroidism. The other two patients displayed overt hypothyroidism. Median TSH of patients with thyroid autoimmunity was 1.77 mIU/L (1.25 - 2.55). Ten patients (9.6%) in this subgroup showed abnormal TSH values, of which six (5.7%) had values below 0.5 mIU/L and 4 (3.9%) had values above 5.0 mIU/L.

Ultrasonography was prescribed to every patient with positive thyroid antibodies; however, only 59 patients (26 females) accepted to undergo the examination, which was performed at a median time of 23 days after the blood tests. Mean thyroid volume was 11.5 mL in males and 9.5 mL in females. Ultrasonographic features of thyroiditis were present in 56 patients (94.9%).

Univariate analysis found that thyroid autoimmunity was positively associated with female sex (p < 0.001) and negatively associated with type 2 diabetes (T2DM) (p = 0.009), but not with hospitalization, ICU admission, respiratory support, or COVID-19 treatment (**Table 1**). Multivariable analysis confirmed the association between thyroid autoimmunity and both female sex and T2DM (see **Table 2**).

**Table 1:** Clinical characteristics of COVID-19 patients according to thyroid autoimmunity status.

	Thyroid autoimmunity		p-value
	No (N=494)	Yes (N=105)	
Age, median (IQR)	65.0 (55.0-73.0)	61.0 (54.0-72.0)	0.30
Gender, n (%)			
Females	132 (26.7)	48 (45.7)	**<0.001**
Males	362 (73.3)	57 (54.3)	
BMI (Kg/m^2^)	26.4 (24.5-29.7)	26.7 (24.6-29.3)	0.89
Hospitalization, n (%)	434 (87.9)	93 (88.6)	0.84
ICU admission, n (%)	50 (10.1)	7 (6.7)	0.27
Respiratory support, n (%)			
None	91 (18.8)	22 (21.4)	0.54
Nasal cannula	115 (23.7)	27 (26.2)	0.59
Venturi mask	54 (11.1)	7 (6.8)	0.19
Reservoir bag	85 (17.5)	16 (15.5)	0.63
CPAP	92 (19.0)	23 (22.3)	0.43
Orotracheal intubation	47 (9.7)	8 (7.8)	0.54
ECMO	1 (0.2)	0 (0.0)	0.64
Comorbidity, n (%)			
Diabetes	76 (15.4)	6 (5.7)	**0.009**
Atrial fibrillation	24 (4.9)	2 (1.9)	0.18
Hypertension	184 (37.2)	32 (30.5)	0.19
IHD	58 (11.7)	9 (8.6)	0.35
Chronic heart failure	22 (4.5)	3 (2.9)	0.46
CKD	46 (9.3)	11 (10.5)	0.71
COPD	20 (4.0)	5 (4.8)	0.74
Autoimmune disease	14 (2.8)	1 (1.0)	0.26
Solid cancer	10 (2.0)	2 (1.9)	0.94
Hematological cancer	9 (1.8)	2 (1.9)	0.95
Liver failure	4 (0.8)	0 (0.0)	0.35
Cerebrovascular disease	12 (2.4)	4 (3.8)	0.43
COVID-19 treatment n (%)			
Lopinavir/ritonavir	215 (43.5)	51 (48.6)	0.34
Hydroxychloroquine	299 (60.5)	62 (59.0)	0.78
Glucocorticoids	169 (34.2)	26 (24.8)	0.061
Antibiotics	312 (63.2)	65 (61.9)	0.81
Tocilizumab	27 (5.5)	2 (1.9)	0.12
Siltuximab	5 (1.0)	3 (2.9)	0.13
Remdesivir	10 (2.0)	2 (1.9)	0.94
Chronic treatment n (%)			
ACE inhibitors	80 (16.2)	16 (15.2)	0.81
ARBs	74 (15.0)	13 (12.4)	0.49
Other antihypertensive drugs	154 (31.2)	23 (21.9)	0.059
Glucocorticoids	19 (3.8)	2 (1.9)	0.33
Oral antidiabetic drugs	56 (11.3)	4 (3.8)	**0.020**
Insulin	14 (2.8)	1 (1.0)	0.26
Oral anticoagulants	43 (8.7)	4 (3.8)	0.090
Antiplatelet drugs	88 (17.8)	21 (20.0)	0.60
PPIs	112 (22.7)	18 (17.1)	0.21

BMI, body mass index; ICU, intensive care unit; CPAP, continuous airway positive pressure; ECMO, extra-corporeal membrane oxygenation; IHD, ischemic heart disease; CKD, chronic kidney disease; COPD, chronic obstructive pulmonary disease; ACE, angiotensin-converting enzyme; ARBs, angiotensin II receptor blockers; PPIs, proton pump inhibitors.

Bold values denote statistical significance at the p < 0.05 level.

**Table 2:** Multivariable analysis of factors correlated with thyroid autoimmunity in the cohort of COVID-19 survivors.

Outcome: Thyroid Autoimmunity	Odds Ratio	Std. Err.	z	P>|z|	[95% Conf. Interval]	
**Age (admission)**	0.9969255	0.0083459	-0.37	0.713	0.9807	1.01342
Sex						
Male	1 (Ref)					
**Female**	**2.01324**	**0.47481**	**2.97**	**0.003**	**1.26808**	**3.19629**
Respiratory support						
No respiratory support	1 (Ref)					
Low need*	0.9432334	0.3005707	-0.18	0.854	0.5051	1.76142
High need*	1.079216	0.3266112	0.25	0.801	0.59635	1.95305
Diabetes mellitus						
No	1 (Ref)					
**Yes**	**0.360492**	**0.1640879**	**-2.24**	**0.025**	**0.14772**	**0.87972**

*Low need: Nasal cannula, Venturi mask; High need: reservoir bag, CPAP, orotracheal intubation ECMO.

Bold values denote statistical significance at the p < 0.05 level.

The control group included 498 patients (320 females, median age 52.7 years). TPOAb were available in 444 patients, TgAb in 373 and both autoantibodies in 325. TPOAb were above the normal range in 37/444 patients (8.3%), TgAb in 33/373 (8.8%) and both antibodies in 14/325 (4.3%) patients.

The sensitivity analysis included 660 subjects (330 patients and 330 controls) matched for age and sex, with a female prevalence of 49.7% for both groups and a median age of 60 (IQR 51-70) in patients and of 59 (47–68) in controls. Positive TPOAb prevalence was higher in patients than in controls (52/330, 15.7% vs. 23/297, 7.7%; p = 0.002), while no difference was observed in positive TgAb prevalence (22/330, 6.7% vs. 20/250, 8%; p = 0.539). Median TPOAb (40 (IQR 31-51) vs. 31 (IQR (27-40)) and TgAb (18 (IQR 14-25) vs. 14 (IQR 14-20)) were within the normal range but significantly higher in COVID-19 patients as compared to controls (both p < 0.001) (**Table 3**).

**Table 3:** Sensitivity analysis comparing 330 COVID-19 patients with 330 pre-pandemic controls.

	COVID-19 Patients	Controls	*p*-value
Female Sex* (n, %)	164/330 (49.7%)	164/330 (49.7%)	
Age* (median, IQR)	60 (51-70)	59 (47-68)	
Positive TPOAb (n, %)	52/330 (15.7%)	23/297 (7.7%)	**0.002**
Positive TgAb (n, %)	22/330 (6.7%)	20/250 (8%)	0.539
TPOAb (U/mL) (median, IQR)	40 (31-51)	31 (27-40)	**< 0.001**
TgAb (U/mL) (median, IQR)	18 (14-25)	14 (14-20)	**< 0.001**

TPOAb, anti-thyroperoxidase antibodies; TgAb, anti-thyroglobulin antibodies.

*Age and sex were matched in cases/controls for the sensitivity analysis.

Bold values denote statistical significance at the p < 0.05 level.

## 4 Discussion

Studies evaluating the impact of SARS-CoV-2 infection on thyroid mainly focused on the alterations of TFTs during the acute phase of the disease, with less evidence about possible long-term effects on thyroid autoimmunity. Our aim was indeed to characterize thyroid autoimmunity and function in the largest cohort of COVID-19 survivors to date.

Most patients had normal TSH levels three months after hospital admission, as already reported in previous studies with smaller cohorts (12–14, 16, 19). Accordingly, the rate of newly diagnosed thyroid dysfunction was comparable to general population (24, 25). This finding seems to rule out a permanent direct damage to the thyroid gland induced by SARS-CoV-2. In this view, the alterations of TFTs in the acute phase of COVID-19 could more probably be secondary to a NTI (16) or a transient, self-limiting, thyroiditis.

The overall prevalence of thyroid autoimmunity in our cohort was 17.5%. Interestingly, the prevalence of positive TPOAb in COVID-19 patients was doubled as compared to controls matched for sex and age (15.7% vs 7.7%). Few authors evaluated thyroid autoimmunity in COVID-19 patients, mostly during the acute phase. Anaya et al. (8) reported an increased prevalence of TPOAb in 120 patients hospitalized for COVID-19 as compared to healthy, pre-pandemic controls (36.7% vs. 20%). Lui et al. (9) identified TPOAb in 20.5% of patients hospitalized for COVID-19; the Authors reevaluated thyroid autoimmunity three months after the admission, reporting a significant increase in TPOAb with 4 out of 82 patients becoming TPOAb positive and an overall prevalence of TPOAb positivity of 25%. The same group confirmed these results in a subsequent study including also asymptomatic COVID-19 patients (19). Our finding of an increased concentration and prevalence of TPOAb in COVID-19 survivors strengthens the hypothesis that SARS-CoV-2 could be able to trigger thyroid autoimmunity; similarly, we found a slightly increased TgAb concentration in patients as compared to controls, though positive TgAb prevalence did not differ between the two groups; TgAb, however, are less useful than TPOAb in predicting thyroid dysfunction (26). Besides, the presence of an actual autoimmune process was consistently confirmed by the evidence of ultrasonographic features of thyroiditis in almost all patients with positive thyroid antibodies.

As already described for other viruses, SARS-CoV-2 may elicit autoimmune conditions through an hyperactivation of both the innate and adaptive immune response (27). Specifically, SARS-CoV-2 may directly trigger thyroid autoimmunity infecting thyroid follicular cells, where ACE-2 receptor is abundantly expressed (28); viral presence has indeed been retrieved in thyroid specimens (29, 30) and reactivity between TPO antigen and SARS-CoV-2 has been demonstrated *in vitro*, favoring the hypothesis of molecular mimicry (31). Alternatively, the hyperinflammatory status caused by severe COVID-19 may induce thyroid damage through the systemic increase of cytokines, unleashing thyroid autoimmunity in genetically predisposed individuals (32). Our findings may suggest that the latter mechanism plays a minor role in triggering thyroid autoimmunity, since the presence of ATD was unrelated with clinical parameters of COVID-19 severity.

In our cohort, thyroid autoimmunity directly correlated with female sex, as expected, and inversely correlated with type 2 diabetes mellitus (T2DM). Diabetes is generally characterized by an impairment of the immune system (33) and diabetic patients with non-severe COVID-19 have a reduced antibodies response to SARS-CoV-2 (34). It is therefore conceivable that diabetic survivors were also characterized by a decrease of autoantibodies against thyroid. Moreover, since during hospitalization in our hospital most diabetic patients were treated with sitagliptin, the immunomodulatory role exerted by the drug (35) may have limited the onset of thyroid immunity.

The strength of our study relies on i) being a large monocentric study, with all patients treated at the same institution and subsequently evaluated by two endocrinologists, and ii) the inclusion of a sensitivity analysis that allowed a direct comparison with age and sex matched controls evaluated before the pandemics.

The main limitations are i) the lack of baseline data about thyroidal status (function and antibodies) of the patients; and ii) not having assessed TSH-receptor antibodies in patients with low TSH. However, it has to be taken into account that routine assessment of thyroid function and autoimmunity is not recommended in the clinical care of acute COVID-19 patients (36).

In conclusion, our study showed that a relatively high proportion of COVID-19 survivors develop both serological and ultrasonographic features of thyroiditis, with only a minority displaying TFTs abnormalities. It is thus possible that the activation of immune response occurring during the acute phase of COVID-19 may induce or precipitate the onset of ATD in some patients. Since the development of thyroid autoimmunity usually precedes the onset of thyroid dysfunction, further longitudinal studies are needed to evaluate thyroid function in a long-term follow-up. Accordingly, the assessment of TPOAb and TFTs could be considered in patients evaluated for long COVID (19), as symptoms of this condition may overlap with those associated with ATD.

## Data availability statement

The raw data supporting the conclusions of this article will be made available by the authors, without undue reservation.

## Author contributions

AR, SC, MR, SB, SV, and RT designed the study. AR and SC evaluated the patients. AR, SI, GC, AG, and TB designed and performed the analyses. AR, FP, and SI drafted the manuscript and prepared figure and tables. All authors contributed to the article and approved the submitted version.
